# Modelling of Brain Deformation After Decompressive Craniectomy

**DOI:** 10.1007/s10439-016-1666-7

**Published:** 2016-06-08

**Authors:** Tim L. Fletcher, Barbara Wirthl, Angelos G. Kolias, Hadie Adams, Peter J. A. Hutchinson, Michael P. F. Sutcliffe

**Affiliations:** 1 Department of Engineering, University of Cambridge, Cambridge, CB2 1PZ UK; 2 Division of Neurosurgery, Department of Clinical Neurosciences, Addenbrooke’s Hospital, University of Cambridge, Cambridge, CB2 0QQ UK

**Keywords:** Neurosurgery, FE analysis, Brain injury

## Abstract

Hyperelastic finite element models, with either an idealized cylindrical geometry or with realistic craniectomy geometries, were used to explore clinical issues relating to decompressive craniectomy. The potential damage in the brain tissue was estimated by calculating the volume of material exceeding a critical shear strain. Results from the idealized model showed how the potentially damaged volume of brain tissue increased with an increasing volume of brain tissue herniating from the skull cavity and with a reduction in craniectomy area. For a given herniated volume, there was a critical craniectomy diameter where the volume exceeding a critical shear strain fell to zero. The effects of details at the craniectomy edge, specifically a fillet radius and a chamfer on the bone margin, were found to be relatively slight, assuming that the dura is retained to provide effective protection. The location in the brain associated with volume expansion and details of the material modeling were found to have a relatively modest effect on the predicted damage volume. The volume of highly sheared material in the realistic models of the craniectomy varied roughly in line with differences in the craniectomy area.

## Introduction

**Figure 1:**
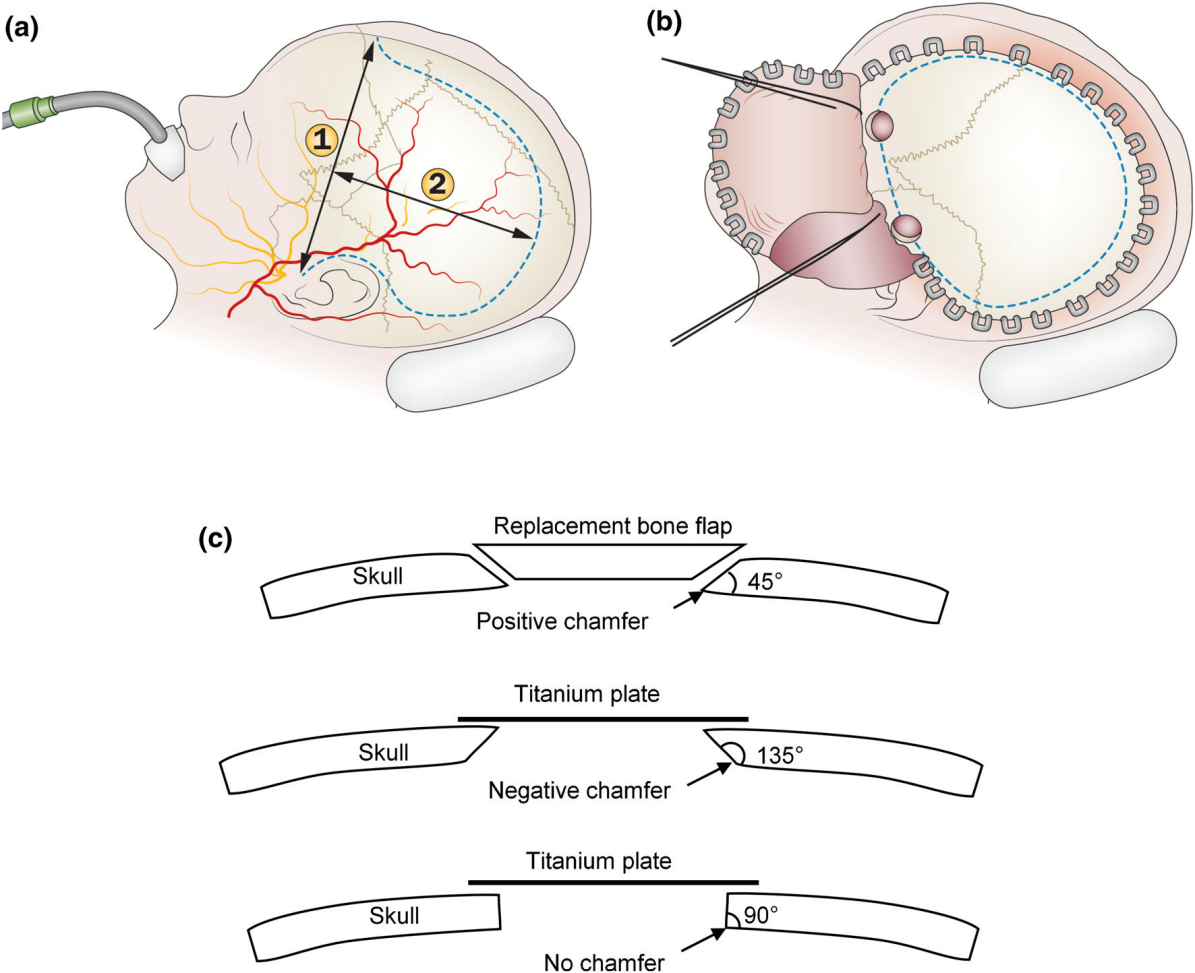
Clinical details of decompressive craniectomy. (a) Schematic of unilateral decompressive craniectomy. The dotted line indicates the skin incision. (b) A skin flap is reflected and the dotted line gives the usual extent of the unilateral craniectomy. (c) Different chamfer options: positive chamfer, negative chamfer, and no chamfer. In this study the chamfer geometry is defined by the chamfer angle, as indicated in the figure. Figures 1a and 1b adapted from Timofeev *et al*.^34^

Traumatic brain injury (TBI) can cause swelling in the brain leading to uncontrolled raised intracranial pressure (ICP), which in turn can cause death or severe brain damage. Hence an important factor in treatment of TBI is reduction of ICP, which can be achieved either by medical or surgical therapies.^20^,^26^ If medical management is unsuccessful then a surgical procedure, decompressive craniectomy (DC), may be used. This procedure is illustrated in Fig. 1. In this operation a portion of the skull is removed, allowing the brain to expand outside the skull and so relieve the pressure.^34^ DC has seen a resurgence of interest in recent years,^19^ but the clinical effectiveness of the treatment remains in doubt.^19^,^21^,^33^,^38^ DC can result in brain deformation and consequent mechanical strains in the brain. Hence it is hypothesised that bioengineering models of DC may provide insight into this applied strain and associated tissue damage.

## Clinical Issues

A number of clinical issues arise which it is hoped that a bioengineering approach can help address.

### Location of Craniectomy

There are two standard forms of DC, bifrontal, and unilateral craniectomies, details of which have been reviewed by Timofeev *et al*.^34^ These differ in terms of the location where part of the skull is removed. Currently there is no consensus on the optimal location of craniectomy, although the unilateral craniectomy is more common.^32^ Surgical decisions on the location of the DC are taken based on the presence of certain features in brain imaging, for example the presence of midline shift (shifting of the brain toward one side), and any swelling present in pre-operative scans.^26^ At present inherent differences in geometry between the choices of craniectomy location are not taken into consideration.

### Size of Craniectomy Opening

Related to the location of the craniectomy, the surgeon has to consider the optimum size of the opening. Evidently a larger opening is likely to lead to a better reduction in ICP, but at a clinical cost of increased risk of adverse effects and complications associated with the surgery.

### Bevel/Chamfer of the Craniectomy

Historically, surgical technique suggests creating a positive chamfer on the bone edge in order to stop the bone flap sinking when it is replaced. Modern cranioplasty tends to be carried out with a titanium or synthetic plate which overlaps the edge of the craniectomy, raising the possibility of using no chamfer, or even a negative chamfer, as illustrated in Fig. 1c. Since Wagner *et al*.^40^ suggest that sharp bone edges can lead to lesions and a poor outcome, changes in bone edge details may have an influence on tissue damage.

## Bioengineering Background

There has been a significant body of work to address brain biomechanics, as reviewed by Goriely *et al*.^16^ Models have considered clinical situations related to hydrocephalus^5^,^29^,^30^ and image guided surgery.^23^,^42^ However, as noted by Goriely *et al*.,^16^ there has been a surprising lack of work considering modeling of DC. A finite element (FE) model of Gao and Ang^15^ uses a poro-elastic material model to simulate changes in ICP and associated deformation after a DC procedure. Both an idealized spherical model and a realistic shape are considered. Results show an encouraging qualitative correlation between the geometry of the deformed brain and clinical observations, and replicate the reduction in ICP associated with the procedure. The effect of increased craniectomy size on increasing deformation and reducing ICP is seen. The authors note that the maximum strains occur around the edge of the craniectomy, which may be associated with tissue damage, but their models are too coarse to capture this effect accurately.

More recently Li and Holst^27^,^39^ have also used a poro-elastic model to simulate the effect of DC. By including ventricular pressure and a “mass effect” fluid source associated with injured tissue, they were able to obtain good agreement between modeled deformations and clinical measurements, and to predict changes in strain associated with performing a DC on the opposite side of the skull to the injured tissue.

In parallel with advances in biomechanics models of tissue deformation, there has been significant work improving the extraction of patient-specific geometries for more accurate modeling of deformation behaviour.^5^,^17^,^24^

The aim of this paper is to follow up on the above modeling work, addressing the clinical questions described above *via* FE modeling. A specific focus of the work is estimating the volume of the brain which might be at risk of damage post operation, by determining the region of brain tissue exceeding a critical strain associated with cell death.^7^ Because of the computational cost of analysing a fully 3D model, a simplified axisymmetric idealized model is first considered. This model is used to perform a parametric study of the changes in geometry that are clinically relevant. However, such a model cannot compare different craniectomy locations. For this, a 3D model of the skull and brain is considered.

## Materials and Methods

### Idealized Cylindrical Model

#### Geometry and FE Implementation

**Figure 2:**
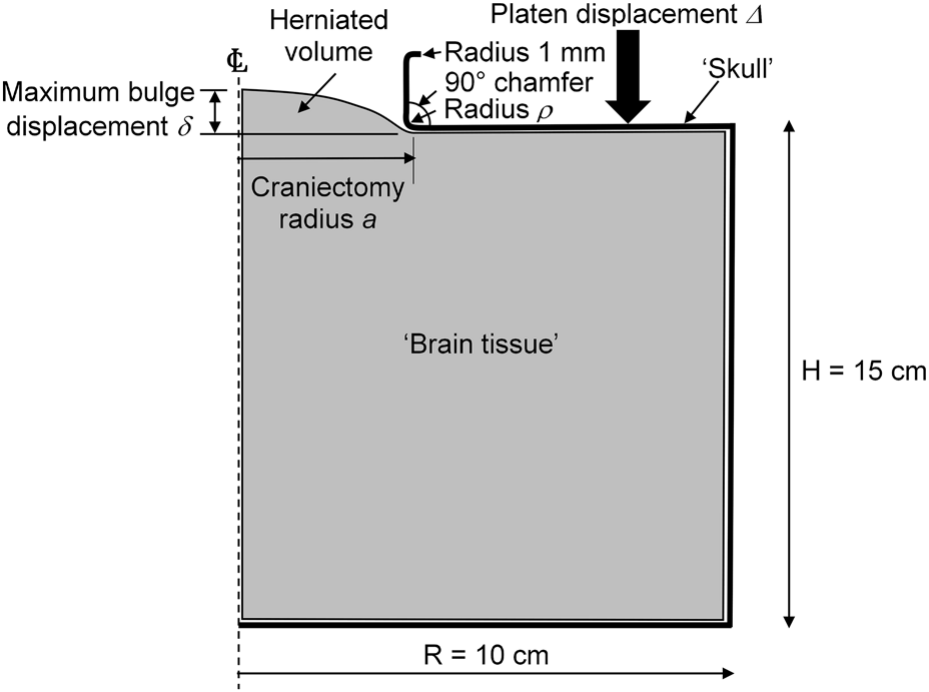
An axisymmetric idealized cylindrical model represents the skull as a cylinder of radius *R* = 10 cm and initial height *H* = 15 cm with a circular craniectomy of radius *a* cut in the top face. Expansion of the brain is simulated by a reduction of the “skull” volume effected by movement downwards of the top platen surface. This gives a corresponding bulge associated with herniation of the brain through the craniectomy opening. The top and side contacts are frictionless, while the bottom of the brain is fixed to the bottom of the skull.

The skull and craniectomy were modeled in the finite element software Abaqus (Dassault Systèmes, France) by an idealized geometry of a cylindrical body containing a circular craniectomy, as illustrated in Fig. 2. Incompressible material inside the “skull” represents the brain tissue. The cylinder has a radius of 10 cm and an initial height of 15 cm, dimensions which are intended to represent the geometry associated with a lateral craniectomy using dimensions appropriate to the skull.^18^ Such a cylindrical geometry has been used in other brain models,^41^ although a spherical geometry would also be appropriate. The volume of the cylinder is reduced by a downwards movement *Δ* of a rigid platen at the top of the cylinder. This platen contains the circular craniectomy of radius *a*. The platen makes frictionless contact with the brain tissue. As the cylinder reduces in volume, brain tissue is forced out of the craniectomy hole. Frictionless contact conditions between the skull and brain tissue on the top face and sides of the cylinder were simulated by constraining brain motion on these faces to be only in the radial and axial directions, respectively, whilst the bottom face was fixed to the skull.

The independent variable in the problem was considered to be the herniated volume of material, which is given by the platen displacement Δ times the cylindrical cross-sectional area *πR* ^2^. The maximum bulge deformation *δ* of the tissue at the herniation is found from the difference in heights between the bottom of the platen and the surface of the brain at the centre of the craniectomy, Fig. 2.

The loading arrangement is intended to represent the clinical situation, where swelling of the brain and an excessive ICP provides the driving force for the herniation of the brain out of the craniectomy opening. Because of the frictionless contact conditions, it is not important where the reduction in cranial volume is generated. Moreover, it is supposed that the details of the strains at the craniectomy will be equivalent for the clinical situation of the enlargement of brain volume, and the modeled situation of a reduction in skull volume. This importance of this assumption, and of the location of the region at which swelling occurs, is addressed in the paper.

A fillet radius *ρ* was included on the inside of the skull at the edge of the craniectomy to simulate the real situation where the dura acts to smooth the corners of the contact between the brain and the skull. The dura is a stiff membrane around the brain. During the DC procedure the dura is circumferentially opened, but the dural opening is smaller than the bone craniectomy opening. This allows the dura to protect the brain from any sharpened edges of the surrounding bone as the brain expands outside the skull. Given that the dura ranges in thickness from 0.3 to 0.8 mm depending on age,^1^ the choice of a 1 mm fillet radius is anatomically relevant. A more sophisticated approach to modeling the meningeal layer is discussed by Yan *et al*.,^43^ with pia and dura mater being modeled by elastic layers separated by a CSF-filled subarachnoid space. The skull thickness is taken as 7 mm, and a fillet radius of 1 mm is also included on the outside edge of the skull. These details are illustrated in Fig. 2.

The model was meshed using axisymmetric 8-noded hybrid elements with reduced integration. The mesh was refined near the craniectomy edge, with a minimum mesh size of 0.05 mm. The resulting model contained approximately 50,000 elements. A mesh refinement study, detailed in Fletcher,^10^ confirmed that this level of refinement gives good accuracy (around 0.2% error in damage volume estimates compared to a further refined mesh).

An implicit analysis was used to solve the problem, including large deformation analysis. Results were analysed using Matlab (Mathworks, USA).

#### Geometric Parameters Considered

An advantage of the idealized model is that it is simple to vary key geometric parameters systematically. Changes in craniectomy diameter, fillet radius, and chamfer angle were considered, varying these parameters in turn from a baseline case with a craniectomy diameter of 10 cm, an internal fillet radius of 1 mm, and with no chamfer (i.e., a chamfer angle of 90°, Fig. 1c).

The craniectomy opening was varied from a minimum diameter of 7 cm to a maximum diameter of 15 cm in 1 cm increments. These are considered to be representative of clinical extremes for a lateral craniectomy.

The importance of the fillet radius *ρ* for the internal edge of the craniectomy is investigated by considering radii of 0.5, 1, and 2 mm.

The effect of craniectomy chamfer is considered by analysing cases with chamfer angles of 45 and 135°, as well as the standard case with an angle of 90° (Fig. 1c).

#### Material Properties

There has been a wealth of work considering material properties for the brain, stretching from work on human and rhesus monkey brain,^8^ through more recent experimental measurements, e.g.,^13^,^28^,^37^ Findings are well covered in various reviews.^4^,^16^,^36^ Specific issues include the anisotropic nature of brain tissue and associated material properties, differences between white and gray matter, and time dependent effects which can be modeled using viscoelastic or poro-viscoelastic material behavior. In preliminary work on FE modeling of DC, Fletcher *et al*.^10^,^11^ explored viscoelastic and poro-viscoelastic models to represent the brain tissue. The conclusion from that study was that the time-dependent material behavior of the brain was not critical to the conditions at the early stages of loading when the maximum extent of the large strain region occurred. Hence in this study we use a simple isotropic hyper-elastic approach to model the material behavior at critical conditions. The importance of this assumption is addressed below, comparing these calculations with a poro-elastic equivalent.

The hyperelastic material model for the brain tissue follows the elastic part of the material model used by Franceschini *et al*.,^14^ with the material response characterized by the Ogden strain energy function relating the strain energy *U* to the deviatoric principal stretches *λ*_1_, *λ*_2_, and *λ*_3_ by

1 $$U = \sum\limits_{i = 1}^{2} {\frac{{2\mu_{i} }}{{\alpha_{i}^{2} }}} \left( {\lambda_{1}^{{\alpha_{i} }} + \lambda_{2}^{{\alpha_{i} }} + \lambda_{3}^{{\alpha_{i} }} - 3} \right)$$

with the parameters *µ*_1_ and *µ*_2_ determining the shear modulus and the parameters *α*_1_ and *α*_2_ determining the strain hardening effect, as given by Table 1. The “Mullins effect” associated with unloading was not included. Incompressible material behavior is assumed.

**Table 1:** Ogden model parameters.^14^

*μ*_1_ (kPa)	*α*_1_	*µ*_2_ (kPa)	*α*_2_
1.044	4.309	1.183	7.736

#### Determination of Potential Damage Region

The deformation induced in the brain after DC may create regions of secondary damage post surgery. In this paper, we report potential damage as a volume of brain tissue that may be at risk of damage, based on shear strain thresholds for brain cell death determined in an *in vitro* model of TBI utilizing stretch of organotypic slice cultures of the rat cortex.^7^ A significant increase in cell death was found at a strain rate of 0.1 s^−1^ for a shear strain of 0.35; hence this is the criterion used for assessing potential damage.

The damage volume was calculated by identifying elements in the axi-symmetric model which exceeded the given threshold shear strain, and summing up the circular regions associated with each element. For larger deformations, a small region at the surface of the brain in the herniated region near the craniectomy edge exceeded the critical strain. This region, which represented less than 5% of the total volume exceeding a critical strain, was neglected as it was considered that damage to this surface region was not likely to be clinically relevant.

### Realistic Model

While the idealized cylindrical model provides a simple way of exploring the effect of some aspects of craniectomy geometry, it cannot address a key issue that the surgeon faces - which location to use for the craniectomy. In this section we describe a 3D model based on a realistic head geometry. Many of the details are common to the idealized model. Specifically the hyperelastic material model described above is again used with an implicit Abaqus solver incorporating large deformations.

#### Geometry and FE Implementation

The realistic brain model is based on an FE mesh produced by Fang and Boas,^9^ taking data from the Collins Brain Atlas.^6^ While this mesh has refinement based on the shape and geometry of the brain and its internal structures, it does not allow for the required refinement at the craniectomy edge. Moreover an analytical model of the skull is needed to create craniectomy openings. Hence the mesh was used to create an analytical model of the skull and brain, which were then re-meshed. This was achieved using Matlab and Pro/E (PTC, USA) in a similar manner to Cheng.^5^ The starting point for the geometry is the mesh of tetrahedral elements generated by Fang and Boas. Cross-sections of the brain and skull at uniformly spaced intervals were then extracted and used to create NURBS spline curves through the intersection of the surface of the brain (or skull) at the given cross section. These splines were connected using the boundary blend tool in pro/E to create a smooth surface and a corresponding solid volume. It was assumed that inclusion of ventricle details would not affect the strains near the craniectomy edge, so the ventricles were modeled as brain tissue. The importance of this assumption is explored below. The geometric models were imported into Abaqus as IGES files for mesh generation. Further details are given in Fletcher *et al*.^10^ An attractive alternative to the above approach would be to use a computer-aided procedure such as that outlined by Hsu *et al*.^17^ to reconstruct patient-specific models from specific computed tomography (CT) images. Details of the craniectomy openings are described in the next section.

Hybrid quadratic tetrahedral elements were used in the model. Mesh refinement was undertaken in the region directly under the bone edge of the craniectomy using partitions and edge seeding. A convergence study was performed on the lateral craniectomy model. A mesh with approximately 150,000 elements produced a percentage error in damage volume when compared to the finest mesh of under 6%, which is considered acceptable. Further details of the geometry, meshing, and FE implementation are given by Fletcher.^10^

Boundary conditions were applied as follows. The skull was fully constrained. The base of the brain was constrained to prevent translations. Frictionless contact conditions were established between the remaining surfaces of the brain and the internal surface of the skull. To alleviate convergence issues associated with this contact condition, the skull was scaled up to create a small (<2 mm) gap between the outer surface of the brain and the inner table. This ensures that there were no overlapping elements between the skull and brain prior to the subsequent expansion of the brain. Moreover a soft exponential contact condition was used to aid convergence, choosing parameters for the clearance and typical pressure of 0.5 mm and 1 kPa, respectively.

Uniform expansion of the brain was generated by changing the temperature of the entire model during the load step, relying on thermal expansion to give the required change in volume. The actual temperature of the model is not part of the solution procedure; the temperature change is solely used as a device to generate the volume change. Due to convergence issues, the herniated volume achieved in these cases was only 22 mL, significantly less than a typical volume clinically.

The extraction of the volume of material region where the shear strain exceeded a critical was performed in Abaqus viewer, using the display group function to isolate and sum the volume of elements which had shear strains greater than the threshold.

#### Craniectomy Openings

Four craniectomy geometries were created in the FE model, the two more common unilateral and bifrontal geometries, and the less common bilateral and bifrontal with a midline bar of bone.

Points defining the location of the craniectomy were placed on the surface of a three-dimensional render of the MRI dataset used to create the FE model by one of the authors (AK), who is a practising neurosurgeon. These points were used to define a cut through the skull in 3D Slicer,^31^ taking care to ensure that the edge of the craniectomy remained approximately perpendicular to the surface of the skull, consistent with clinical practice. As with the idealized model, a fillet radius of 1 mm was added to all internal bone edges of the craniectomy.

**Figure 3:**
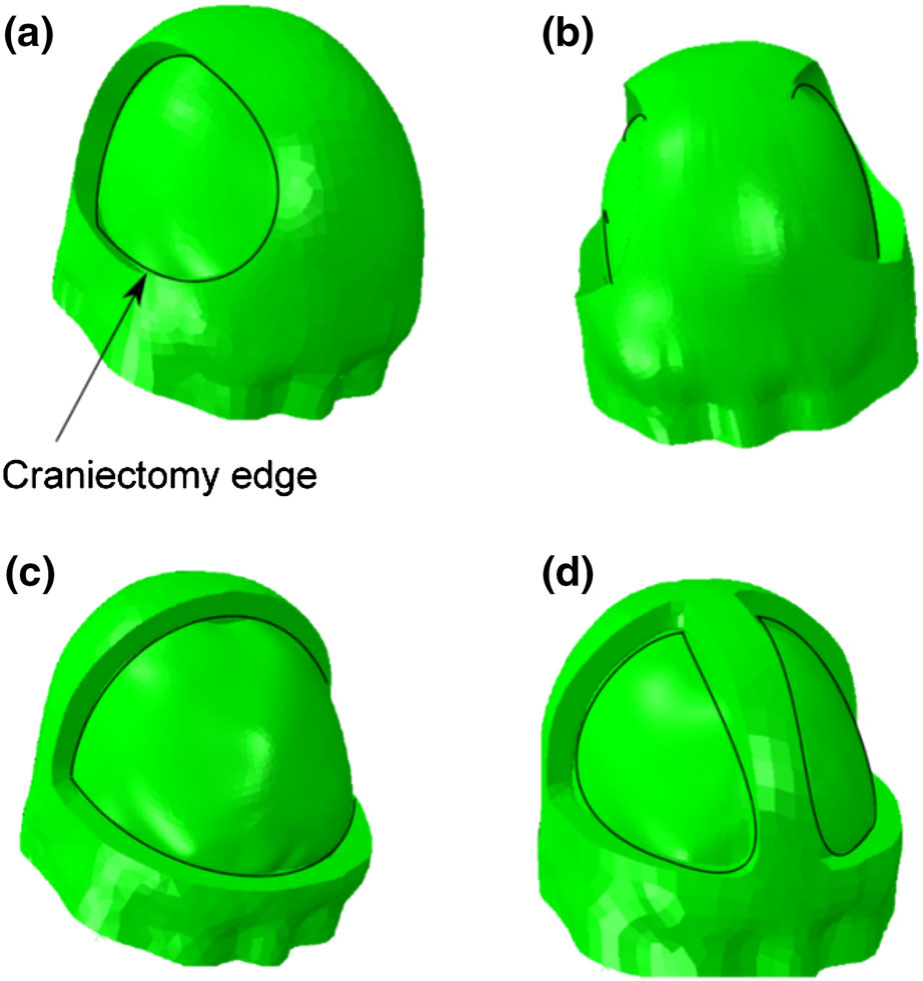
A range of craniectomy geometries are considered in the realistic model: (a) unilateral, (b) bilateral, (c) bifrontal, (d) bifrontal with midline bar. Each of the craniectomy openings have been generated based on points placed on a rendering of the MRI dataset by a practising neurosurgeon (AK).

The locations of the craniectomy openings are illustrated in Fig. 3. The bifrontal and unilateral openings followed those in Kolias *et al*.^25^ Table 2 compares the craniectomy surface areas for the four procedures.

**Table 2:** Comparison of different craniectomy geometries.

Geometry	Unilateral	Bilateral	Bifrontal	Bifrontal with a midline bar
Surface area of opening (cm^2^)	98	190	260	180
Maximum bulge displacement (mm)	5.2	2.8	3.4	4.0
Volume exceeding a shear strain of 0.15 (mL)	5.2	0.25	0.27	2.2

### Assessment of Geometric and Material Modeling Assumptions

A potential limitation of the idealized model is the absence of internal features including the ventricle, and the simplified way in which the expansion has been modeled by a reduction in skull volume. To investigate these effects, further FE models were developed, taking a 10 cm diameter craniectomy and uniformly expanding the brain tissue, rather than reducing the “skull” volume. To explore the effect of ventricles on the deformation, a “ventricle” of approximately elliptical cross section, with initial volume of 29 mL and diameter of 6.4 cm was created at the centre of the brain. This ventricle was ascribed a shear modulus 1/30th of the surrounding material. To understand how the deformation depends on the location where expansion occurs, a model was considered where the central ventricle was given an expansion corresponding to 50% of the total 85 mL herniation, with the remaining 50% contributed by the remaining brain tissue.

Another potential limitation of the work is the simplified hyper-elastic material used. To quantify the sensitivity of the results to the inclusion of time-dependent deformation behavior associated with poro-elastic effects, a poro-elastic FE model has been developed for the idealized case, following the work of Fletcher *et al*.^11^ The ventricle was modeled as a roughly ellipsoidal cavity with an internal pressure of 20 mmHg, relative to the pressure around the boundaries of the brain. The hyper-elastic model detailed in Table 2 was used for the brain tissue, with the addition of some compressibility *via* a bulk modulus term *D*_1_. Values of *D*_1_ equal to 7.2 and 93 kPa were used, corresponding to effective Poisson’s ratios of 0.496^14^ and 0.45,^27^ respectively. A value of permeability of 2.42 × 10^−10^ m/s was used.^14^ Since the ventricle pressure was insufficient to achieve the target 85 mL herniation, an additional global expansion of the brain tissue was also applied. The effect of loading time was investigated by applying the ventricle pressure and expansion changes over either 100 s or 12 h.

### Evaluation of Deformation from Patient Data

To make a comparison with the idealized geometry modeling, in-plane deformations were derived from clinical CT X-ray images pre- and post-op. To obtain the clinical deformation field, the pre-op CT scan of a patient with TBI was mapped to the post-op CT scan after DC using the BRAINSDemonWarp module^22^ in 3D Slicer.^31^

All CT scans were acquired during routine clinical care. Anonymised clinical data were collected in the course of the RESCUEicp study (Randomised Evaluation of Surgery with Craniectomy for Uncontrollable Elevation of intracranial pressure trial—ISRCTN66202560), RESCUE-ASDH study (Randomised Evaluation of Surgery with Craniectomy for patients Undergoing Evacuation of Acute Subdural Haematoma—ISRCTN87370545), and from clinical audit of patient care in the Neurosciences Critical Care Unit/Neurosurgical Unit of Addenbrooke’s Hospital. Ethical approval for the RESCUEicp study has been obtained from the UK Multi Centre Research Ethics Committee (Eastern Region) and the clinical audit has been registered and approved by the Clinical Audit Department, Addenbrooke’s Hospital. The RESCUE-ASDH study acquired ethical approval from the National Research Ethics Service (North West Region). All records/information were anonymized and de-identified prior to analysis.

Data relating to the study are available at Cambridge University’s online repository at http://dx.doi.org/10.17863/CAM.14.

## Results

### Cylindrical Model Results

**Figure 4:**
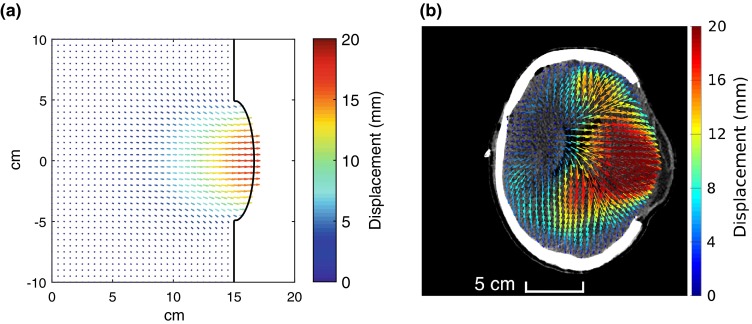
Qualitative comparison of modeling and clinical displacement fields: (a) idealized geometry with craniectomy diameter 2*a* = 10 cm, for a herniation volume of 85 mL, (b) clinical data for the in-plane displacement between pre- and post-op CT images for a patient undergoing a unilateral craniectomy. The pattern of deformation is qualitatively similar, except where affected by the details of the ventricle expansion.

Figure 4 gives a qualitative comparison between the deformation field predicted by the cylindrical model, for the baseline case with a craniectomy diameter of 10 cm, and in-plane deformations obtained from clinical CT X-ray images pre- and post-op. The general pattern of deformation is qualitatively similar in the theoretical and clinical results in the top half of the figures. At the bottom of the figures, expansion of the ventricle post-op has affected the clinical deformation pattern. This issue is returned to below.

#### Effect of Herniated Volume and Craniectomy Size

**Figure 5:**
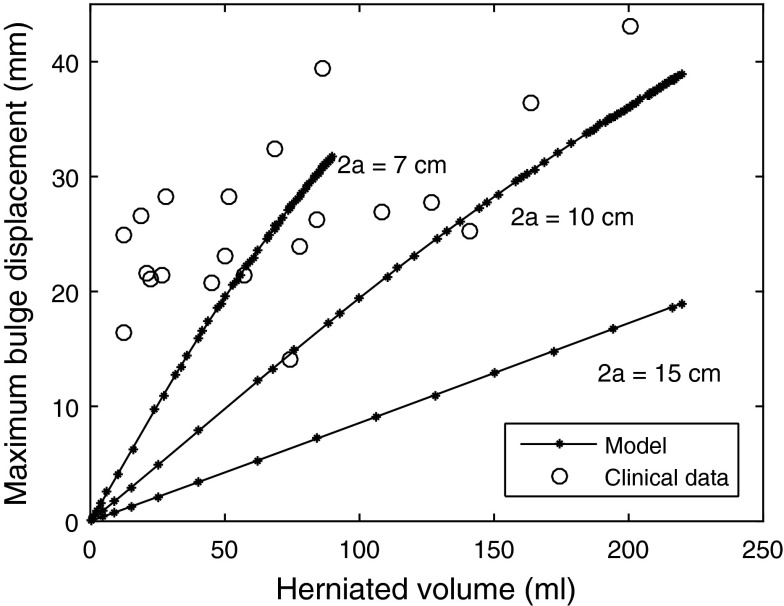
The effect of herniated volume and craniectomy diameter 2*a* on the maximum bulge displacement *δ*, for the idealized cylindrical model. The displacement increases roughly linearly with increasing volume. Clinical data for lateral and bifrontal craniectomies are taken from Fletcher *et al*.,^12^ showing a reasonable consistency with predictions.

Figure 5 illustrates results for the idealized cylindrical model showing the relationship between herniated volume and maximum displacement of the herniated tissue, for three craniectomy diameters of 7, 10, and 15 cm. The displacement increases roughly linearly with increasing volume. The larger the craniectomy area, the smaller the displacement for a given herniated volume. The proportionality of bulge with volume would hold for self-similar deformation, but with the large strain deformation included, it is not evident that this will be the case. This linear response is reasonably consistent with measurements from a set of clinical data with lateral and bifrontal craniectomies,^12^ shown as circles on Fig. 5. These results are for craniectomy areas in the inter-quartile range from 97 to 125 cm^2^, where the area was characterized by the height times the width of the craniectomy.

**Figure 6:**
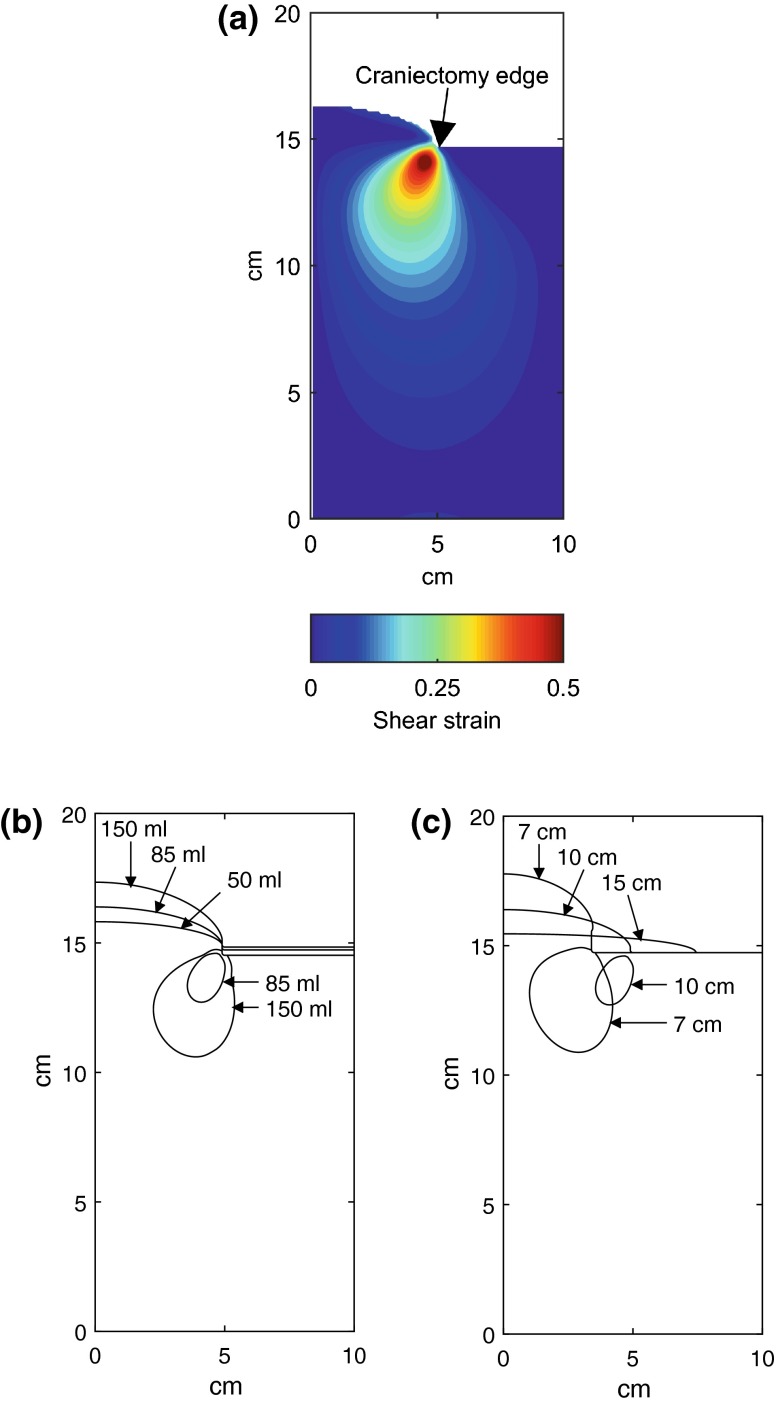
Herniated brain profiles and shear strains for the idealized cylindrical model with a fillet radius *ρ* = 1 mm, and no chamfer. (a) Shear strain field with a craniectomy diameter 2*a* of 10 cm and a herniated volume of 85 mL. The peak shear strains occur at the edge of the craniectomy opening. (b) Effect of herniated volume on brain profile and contours of shear strain equal to 0.35, for a craniectomy diameter 2*a* of 10 cm. The region of high shear strain remains at the edge of the craniectomy opening and increases with increasing hernation volume. There is no region exceeding a shear strain of 0.35 for a herniated volume of 50 mL. (c) Effect of craniectomy diameter on brain profile and contours of shear strain equal to 0.35, for a herniated volume of 85 mL. The extent of the region of high shear strain diminishes as the craniectomy opening increases. There is no region exceeding a shear strain of 0.35 for a craniectomy diameter of 15 cm.

The shear strain within the brain tissue is plotted in Fig. 6a for the baseline case with a craniectomy diameter of 10 cm, a fillet radius of 1 mm and a herniated volume of 85 mL. The centreline of the axisymmetric model is at the left of the figure. As expected, the region of most intense shear is at the craniectomy edge. Because of the 1 mm radius on the bone margin at this position, the maximum stress lies somewhat below the surface.

Figure 6b shows the changes with increasing herniated volume in the shape of the surface profile of the herniated brain and in the contour of shear strain equalling 0.35 (material inside this contour exceeds this value). Results are again taken for the baseline case of a craniectomy of diameter 10 cm. The range of herniated volumes shown from 50 to 150 mL is in the clinical range for a lateral craniectomy. There is a significant growth in the region of potential damage as the herniated volume increases, with the region extending deeper into the brain tissue.

The effect of craniectomy diameter on the shape of the herniated brain surface and the contours of critical strain are shown in Fig. 6c. As the craniectomy area increases, so the contour of critical strain reduces in size, extending less far into the brain. Although the cross sectional area of the damage region is about five times greater for the 7 cm craniectomy, compared with the 10 cm diameter craniectomy, the corresponding volume differs only by a factor of about three on account of the larger peripheral length with the larger craniectomy.

**Figure 7:**
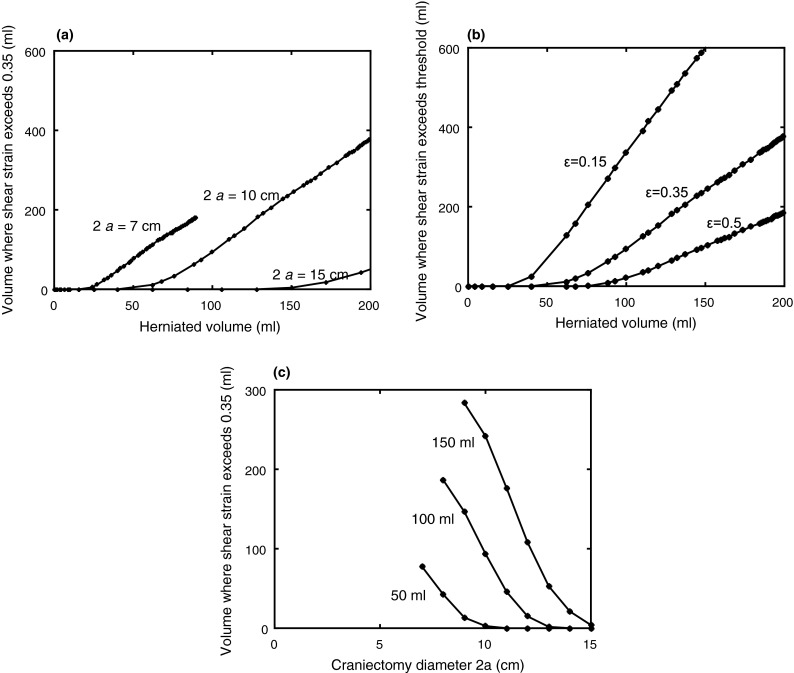
Idealized model geometry results for the volume of material exceeding a threshold shear strain. (a) Effect of herniated volume and craniectomy diameter on the volume exceeding a threshold strain of 0.35. There is a threshold herniated volume below which the region of high shear strain falls to zero. Above the threshold herniated volume, the region of high shear strain increases with increasing herniated volume. (b) Effect of herniated volume and threshold strain level on the volume exceeding the strain threshold, for a craniectomy diameter of 10 cm. The volume of material above a critical shear strain increases significantly with the change in critical shear strain. (c) Effect of craniectomy diameter 2*a* on the volume of material exceeding a threshold shear strain of 0.35. There is a significant reduction of material exceeding a critical shear strain with increasing craniectomy area.

The evolution of the damage volume with herniated volume is shown in Fig. 7a for three craniectomy diameters of 7, 10, and 15 cm, choosing a critical shear strain of 0.35 as suggested by the results of Elkin and Morrison.^7^ Figure 7b compares the growth of damage volume with herniated volume for three critical shear strain values of 0.15, 0.35, and 0.5, for the baseline case of a 10 cm craniectomy. The volume of material exceeding the critical strain only starts to rise after a critical herniated volume is reached, with this critical value increasing with increasing craniectomy diameter. As long as the herniated volume exceeds this threshold, there is a significant benefit in increasing the craniectomy diameter. A reduction in the critical shear strain, needless to say, increases the volume of material at risk of damage. But the trends for changes of damaged volume with herniated volume are similar at the different levels of critical shear strain.

Results for the effect of craniectomy diameter on damage volume are summarized in Fig. 7c, for herniated volumes of 50, 100, and 150 mL and at a critical strain of 0.35. As the required herniated volume increases, it becomes increasingly helpful to increase the craniectomy diameter.

#### Craniectomy Bone Edge Details

**Figure 8:**
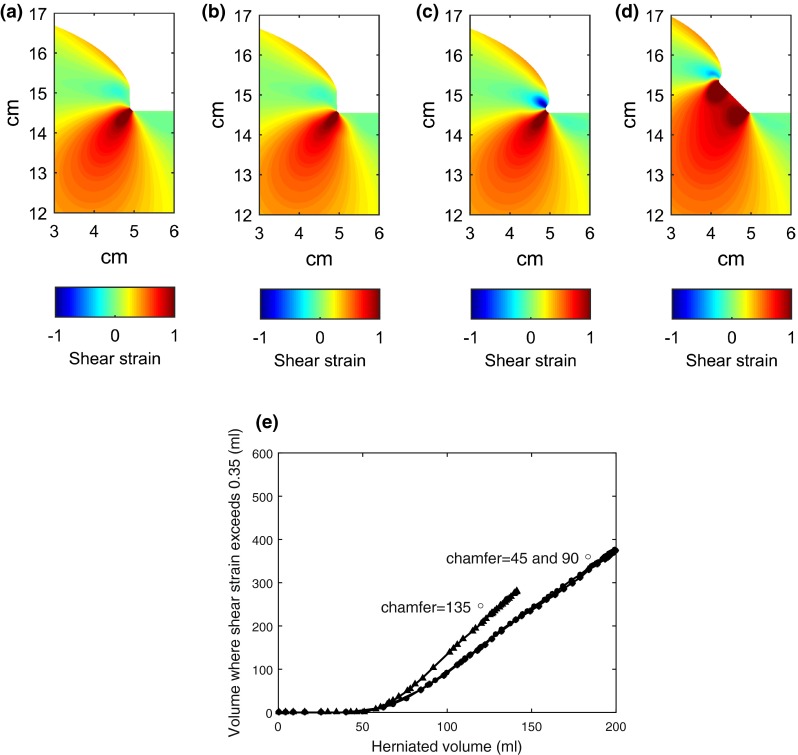
Effect of craniectomy edge details on the idealized geometry shear strain predictions. (a) to (d) Local shear strain distribution for a herniated volume of 140 mL: (a) base line case with 1 mm fillet radius, (b) fillet radius *ρ* = 0.5 mm, (c) positive chamfer of 45°, (d) negative chamfer of 135°. (e) Effect of chamfer on the relationship between herniated volume and the volume of material exceeding a shear strain of 0.35, for a craniectomy diameter of 10 cm. There are differences in the details of the shear strain distribution local to the surface of the chamber, but the sub-surface strain field is little affected by the chamfer details, resulting in only a modest effect on the volume of material exceeding the critical shear strain.

Figures 8a and 8b compare the shear strain at the craniectomy edge for fillet radii of 1 and 0.5 mm, respectively, with a herniated volume of 140 mL. Note that, in these plots, only the region close to the craniectomy edge has been plotted and the scales for shear strain have been expanded to include positive and negative shear. It is the magnitude of the shear which will determine damage. There is only a very slight effect of fillet radius on the strain field. The volume of material exceeding a threshold strain of 0.35 differs by only 8% between the 0.5 and 2 mm fillet radii, for a herniated volume of 150 mL. These results suggest that, as long as the dura is present to smooth off the edge of the skull, the fillet radius will not make a significant difference to potential damage within the brain tissue.

Figures 8a–8d compare shear strain distributions for the 90°, 45°, and 135° chamfers, respectively. Small differences at the surface are seen, with the worst case being the 135° chamfer where the two changes in contact angle provide extra scope for raised shear strains, while there is some heightened shear strain for the positive 45° chamfer at the craniectomy edge. However the bulk of the tissue below the surface suffers similar shear strains, irrespective of the chamfer angle. The corresponding relationships between the herniated volume and the volume exceeding the critical shear strain of 0.35 are plotted on Fig. 8e. Differences are relatively small, with the greater damage volume for the negative 135° chamfer being partially explained by the smaller effective opening that this geometry presents at large deformations.

### Assessment of Geometric and Material Modeling Assumptions

**Figure 9:**
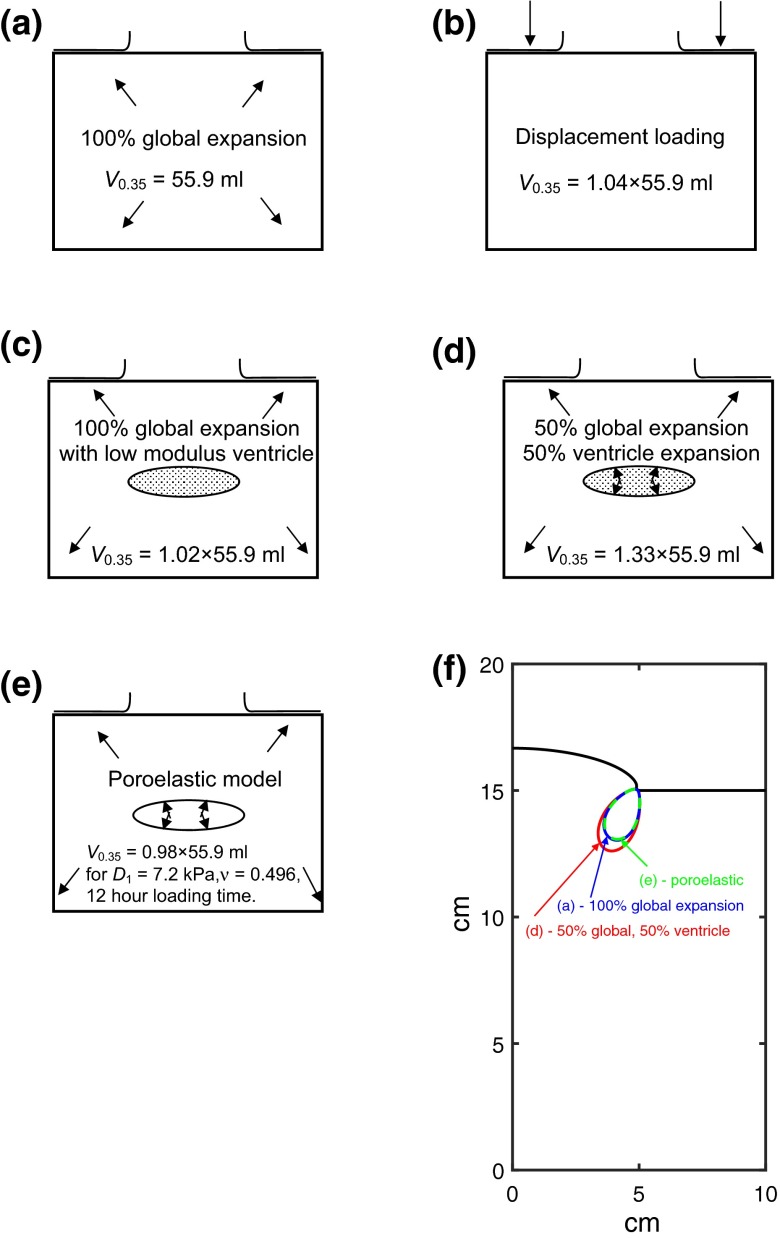
Effect of modeling assumptions on volume of material exceeding a threshold strain, for a herniation volume of 85 mL. (a)–(e) different modeling scenarios used and volume *V*_0.35_ of material exceeding a threshold strain of 0.35 (*V*_0.35_ is expressed as a multiple of that for case (a)), (f) contours of shear strain equal to 0.35, for selected modeling cases. There is only a modest effect of modeling assumptions on the volume of material exceeding the threshold strain.

Figure 9 compares different cases exploring the effect of geometric and material modeling assumptions on the volume of material exceeding a critical shear strain of 0.35. In each sub-figure the volume is expressed as a multiple of that for the baseline case, Fig. 9a. A comparison of the model with uniform expansion of the brain tissue (Fig. 9a) with the standard model in which the skull is reduced in volume (Fig. 9b), shows a nearly identical pattern of strains with only a 4% change in the volume of material exceeding the critical shear strain. The case with a low modulus “ventricle” at the centre of the brain, Fig. 9c, gives a 2% increase in volume exceeding the critical shear strain, compared to the uniform expansion model of Fig. 9a, confirming that volume-conserving changes in deformation behavior of the ventricle are not critical to the shear strain distribution at the craniectomy edge. Figure 9d shows the case where the central ventricle was given an expansion corresponding to 50% of the total 85 mL herniation, with the remaining 50% contributed by the remaining brain tissue. The significant local strains in the ventricle region do in this case affect the surface strains, with a 33% increase in the volume of the high strain region. The corresponding contours of shear strain equal to 0.35 for the two cases of 100% global expansion, and 50% ventricle, 50% global expansion are compared in Fig. 9f, showing how the shape of the affected region is enlarged with the model including this enhanced ventricle expansion.

Figure 9e illustrates the model used to quantify the effect of including a poro-elastic analysis. With an effective Poisson’s ratio of 0.496 derived from the experiments of Franceschini *et al*.^14^ and a loading time of 12 h, the resulting volume of material exceeding the critical shear strain of 0.35 fell by 2%, relative to the hyper-elastic case with 100% global expansion (i.e., Figure 9a). The corresponding contour of shear strain equal to 0.35 is given in Fig. 9f, showing an almost identical shape to the standard case of Fig. 9a without poro-elastic material behavior. The poro-elastic case with the same Poisson’s ratio but a shorter loading time of 100 s showed a 1% reduction in volume compared to the standard case. Using a Poisson’s ratio of 0.45 suggested by Li and van Holst^27^ yields damage volumes 1 and 22% less than the standard case of Fig. 9a, for loading times of 100 s and 12 h, respectively.

### Realistic geometry results

**Figure 10:**
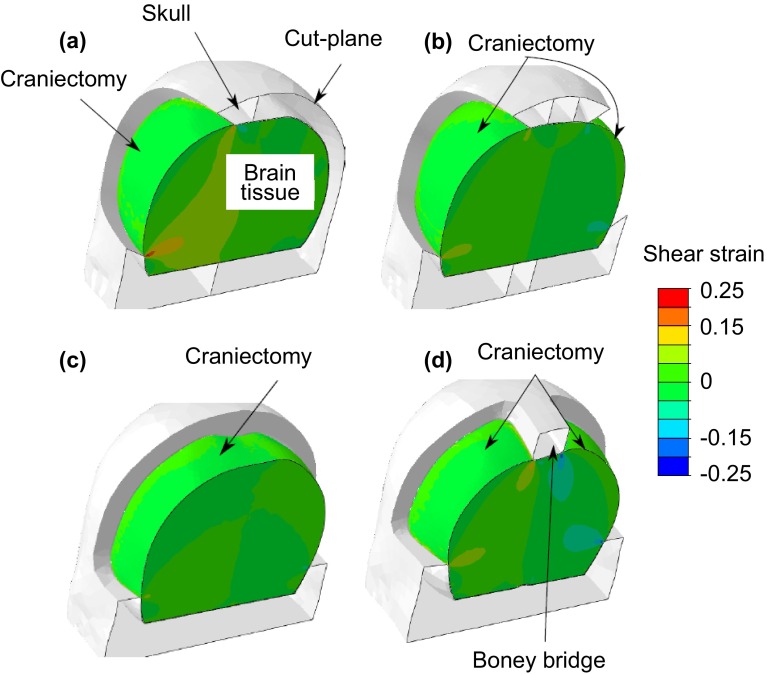
Shear strain distribution on slices through the different craniectomy geometries: (a) unilateral, (b) bilateral, (c) bifrontal, (d) bifrontal with midline bar. The details of the craniectomy geometry have a significant effect on the shear strain distribution.

Figure 10 compares the shear strains developed for the four different realistic craniectomies considered. Recall that the herniated volume in these cases is only 22 mL due to convergence issues, significantly less than a typical volume clinically, so that the shear strains are on the low side. Nevertheless, the results from the idealized geometry model show that trends seen with small herniated volumes extrapolate to larger deformations. Figure 10 shows distinct differences between the craniectomy types. The unilateral craniectomy has the highest strains and the largest regions of high strain under the craniectomy edge. The unilateral craniectomy is followed in severity of shear strain by the bifrontal with the midline bar and then the bilateral. Finally the bifrontal craniectomy shows very little shear strain when plotted on the same strain scale as the other craniectomy openings. This pattern follows the size of the craniectomy opening given in Table 2.

Table 2 further quantifies the differences between the craniectomy locations, tabulating the maximum bulge deformation and volume of material exceeding a shear strain of 0.15 for the four geometries. This lower critical shear strain, as compared with the value of 0.35 used for the idealized model, is chosen to account for the reduced herniation volume in these analyses. The smaller opening of the unilateral DC contributes to the largest bulge deformation and region of critical shear. The next worst is the bifrontal with the midline bar, with the bilateral and bifrontal giving the smallest volumes exceeding a critical shear.

## Discussion

Although the cylindrical model does not have the same geometry as a skull, the trends seen between geometric parameters and the region at risk of damage are expected to apply to the clinical situation, albeit with quantitative differences associated with a realistic geometry.

Results from the idealized model confirm the expectation that a larger craniectomy opening is potentially less likely to damage tissue due to high strains present after the DC procedure. However, for a given herniation volume, there is a threshold area beyond which the volume of damage falls to zero, so that a further increase in craniectomy area would not be predicted to give a significant improvement in damage area. For example, for clinically typical herniation volumes of 50 and 100 mL, this threshold diameter is around 10 and 13 cm, respectively, Fig. 7c. This biomechanics approach to potential damage needs to be balanced with clinical issues associated with larger craniectomy areas, including complications associated with infection, more challenging cranial reconstruction and a potentially higher risk of hydrocephalus.^26^

The qualitative comparison of deformation between the idealized model and a clinical case, Fig. 4, highlights a potential limitation of the idealized model, with the absence of internal features including the ventricle, and the simplified way in which the expansion has been modeled by a reduction in skull volume. However the results of Fig. 9 exploring these effects show that modeling brain expansion using a reduction in skull volume does not introduce significant errors into the model. The effect of a soft ventricle on deformation at the craniectomy edge is also found to be relatively slight for the baseline case considered, confirming that volume-conserving changes in deformation behavior of the ventricle are not critical to the shear strain distribution at the craniectomy edge. However changes in the location of the region of brain expansion were seen to affect results, with a 33% increase in the volume of the high strain region where 50% of the expansion was assigned to the central ventricle. The simplified ellipsoidal ventricle shape used probably over-estimates the effect of ventricle expansion on deformations elsewhere, since clinically the complex ventricle shapes allow collapse and expansion without necessarily leading to the strains in the surrounding material associated with the idealized shape used. Nevertheless this result illustrates that further work modeling the locations at which brain expansion occur, combined with a realistic model of ventricle expansion, would be valuable in patient-specific modeling of strain distribution.

Another potential limitation of the work is the simplified hyper-elastic material used. The approach used here has been supported by the preliminary FE study of idealized DC geometry by Fletcher *et al*.,^11^ who concluded that the time-dependent material behavior of the brain was not critical to the conditions at peak load and strain. There are two aspects of poro-elastic models which can be dealt with separately, volume changes associated with DC and time-dependent deformation associated with fluid flow. The first point concerns the nature of the change in volume of the brain tissue associated with DC. Here CSF and ICP play a role *via* increasing the ventricle volume. But clinical observations show that such ventricle volume changes do not fully account for the herniated volume observed. Hence there is a need for a significant additional mechanism of volume increase, which in von Holst and Li^39^ is provided by a “mass effect,” represented by a fluid source. If this mass effect were associated with interstitial edema it might be helpful to model this additional volume increase using a poro-elastic flow of CSF. But other mechanisms for a change in brain volume are an increase in cell volume associated with cell damage, an increase in vascular volume, and a change in volume associated with brain herniation downwards through the foramen magnum. None of these other mechanisms are associated with CSF movement so that a poro-elastic model associated with CSF flow would not be appropriate in these cases. The review of Unterberg *et al.* 35 concludes that interstitial edema is rare in acute-phase TBI, with cytotoxic and vasogenic effects being dominant. For this reason, we conclude that, although changes in ventricular volume associated with ICP are relevant to brain volume changes in acute-phase TBI, it is not necessary to model additional volume changes associated with cytotoxic and vasogenic effects using a poro-elastic model. Instead, for this aspect of the biomechanics, the approach adopted of specifying a volume increase in the brain tissue is an appropriate way to model this additional volume increase. As noted above, changes in the location of volume expansion, particularly associated with ventricle enlargement, do have some influence on the strain field around the craniectomy. Hence further work would be helpful to determine the distribution of brain expansion throughout the brain associated with DC, exploring effects associated with physiological changes in the tissue.

In any case the results of Fig. 9 show that time-dependent porous flow does not play a large role in the critical deformations, and that the hyper-elastic material formulation used is a reasonable model of the process.

An investigation of the effect of craniectomy edge fillet radius and chamfer suggest that, as long as the dura provides an effective protection from the sharp edge of the skull, the details of the craniectomy edge are not critical to the area of potential tissue damage. There are relatively small changes in shear strain local to the craniectomy edge, but these are over-shadowed by the much larger volumes of potentially damaged material sub-surface. There was no significant change in deformation associated with the skull fillet radius changing from 0.5 to 2 mm. In terms of chamfer at the craniectomy edge, the best situation was seen to be the 90° chamfer, corresponding to a cut perpendicular to the skull surface. The sharp 45° chamfer gave a small region of heightened shear strain compared to the 90° chamfer, albeit the change was relatively small. The worst case was the 135° chamfer which, although providing a more gradual corner at the inner surface, resulted in a higher area of shear strain and effectively reduced the craniectomy area.

In general the realistic geometry results correlate with the results of the idealistic model, with the larger craniectomy areas giving smaller deformation and smaller potential damage regions. An exception to this is the bifrontal with the midline bar geometry, which had a somewhat higher volume of high shear strain than might be expected given its area. This is associated with the constraint of the bony bridge over the sagittal sinus which blocks the expansion of the brain in the region of largest deformation.

The strain threshold used in the present study was based on an *in vitro* model of TBI utilizing stretch of organotypic slice cultures of the rat cortex.^7^ These are currently the best available data, however they do not take into account different viscoelastic properties of cortical gray matter (GM) and white matter (WM). Studies have suggested that GM is significantly softer and less viscous than WM, therefore future models should aim to include these properties when modeling deformity of the brain.^3^ New techniques such as magnetic resonance elastography can potentially offer more insight into the viscoelastic properties of brain tissue and further refine strain modeling.^2^

Due to convergence issues, the expansion of the brain for the realistic model was restricted to 22 mL, on the low side compared with typical clinical values in the range 20–140 mL.^12^ Nevertheless, the results from the idealized model show that deformation and damage volume trends established at these lower herniation volumes carry through to the larger more clinically appropriate values. Use of a lower critical strain to characterize damage in these realistic analyses can effectively compensate for this issue.

## Conclusions

Hyperelastic finite element models, with either an idealized cylindrical geometry or with realistic craniectomy geometries, have been used to explore clinical issues relating to DC. The potential damage in the brain tissue has been estimated by calculating the volume of brain tissue exceeding a critical shear strain. The following conclusions can be drawn:

- the maximum bulge displacement is predicted to increase roughly linearly with increasing herniated volume, also decreasing with increasing craniectomy area for a given herniated volume;
- the region of maximum shear strain is below the surface of the brain, increasing in extent with increasing herniation volume and decreasing craniectomy diameter;
- for a given herniation volume, there is a threshold craniectomy diameter at which the volume of potentially damaged tissue falls to zero;
- the effect of details at the craniectomy edge (fillet radius and chamfer) are relatively small, assuming that the dura is retained to provide effective protection;
- the volume of highly sheared material in the realistic models of the craniectomy varies roughly in line with changes in craniectomy area, although the case of the bifrontal with midline bar is notable for giving somewhat more volume of damaged material than would be expected from the craniectomy area;
- while the above models provide biomechanical guidance for changes in DC geometry, these will need to be balanced with clinical issues.
